# Human Salivary Histatin-1 Promotes Osteogenic Cell Spreading on Both Bio-Inert Substrates and Titanium SLA Surfaces

**DOI:** 10.3389/fbioe.2020.584410

**Published:** 2020-10-23

**Authors:** Wei Sun, Dandan Ma, Jan G. M. Bolscher, Kamran Nazmi, Enno C. I. Veerman, Floris J. Bikker, Ping Sun, Haiyan Lin, Gang Wu

**Affiliations:** ^1^The Affiliated Stomatology Hospital, Zhejiang University School of Medicine, Hangzhou, China; ^2^Key Laboratory of Oral Biomedical Research of Zhejiang Province, Hangzhou, China; ^3^Department of Oral Biochemistry, Academic Centre for Dentistry Amsterdam, University of Amsterdam and Vrije Universiteit Amsterdam, Amsterdam, Netherlands; ^4^Savaid Stomatology School, Hangzhou Medical College, Hangzhou, China; ^5^Department of Oral Implantology and Prosthetic Dentistry, Academic Centre for Dentistry Amsterdam, University of Amsterdam and Vrije Universiteit Amsterdam, Amsterdam, Netherlands; ^6^Department of Oral and Maxillofacial Surgery/Pathology, Amsterdam UMC and Academic Center for Dentistry Amsterdam, Vrije Universiteit Amsterdam, Amsterdam Movement Science, Amsterdam, Netherlands

**Keywords:** salivary, peptide, osteogenic cells, cell spreading, osteoconductivity

## Abstract

Promoting cell spreading is crucial to enhance bone healing and implant osteointegration. In this study, we investigated the stimulatory effect of human salivary histatin-1 (Hst-1) on the spreading of osteogenic cells *in vitro* as well as the potential signaling pathways involved. Osteogenic cells were seeded on bio-inert glass slides with or without the presence of Hst1 in dose-dependent or time-course assays. 1 scrambled and 6 truncated Hst1 variants were also evaluated. Cell spreading was analyzed using a well-established point-counting method. Fluorescent microscopy was adopted to examine the cellular uptake of fluorescently labeled Hst1 (F-Hst1) and also the cell spreading on sandblasted and acid etched titanium surfaces. Signaling inhibitors, such as U0126, SB203580, and pertussis toxin (PTx) were used to identify the potential role of extracellular-signal-regulated kinase, p38 and G protein-coupled receptor pathways, respectively. After 60 min incubation, Hst1 significantly promoted the spreading of osteogenic cells with an optimal concentration of 10 μM, while truncated and scrambled Hst1 did not. F-Hst1 was taken up and localized in the vicinity of the nuclei. U0126 and SB2030580, but not PTx, inhibited the effect of Hst1. 10 μM Hst1 significantly promoted the spreading of osteogenic cells on both bio-inert substrates and titanium SLA surfaces, which involved ERK and p38 signaling. Human salivary histatin-1 might be a promising peptide to enhance bone healing and implant osteointegration in clinic.

## Introduction

Large-volume bone defects (LVBD) can result from various diseases, such as congenital malformation, trauma, infection, inflammation and cancer (Hak, 2007; Mcallister and Haghighat, 2007; Chiapasco et al., 2009). Due to limited regenerative capacity of bone tissue, LVBD usually causes delayed repair or non-union—a permanent failure of healing, which may severely compromise aesthetics and musculoskeletal functions of patients. Hitherto, the repair of LVBD is still challenging in the fields of oral implantology, maxillofacial surgery and orthopedics. Autologous bone grafts are still regarded as the gold standard treatment for LVBD (Kneser et al., 2006). However, their applications are hindered by donor-site morbidity and limited availability. As alternatives to autologous bone, a large variety of medical devices, such as allografts, xenografts, synthetic materials have been developed and applied (Laurencin et al., 2006; Kumar et al., 2013). Most of these medical devices bear intrinsic osteoconductivity—the ability to support the attachment, spreading, migration and ingrowth of osteogenic cells within bone substitues (Albrektsson and Johansson, 2001). However, in general their osteoconductivity appears too limited to result in a completely osseous healing of LVBD. Besides, in dental implantology, continuous efforts are being made to enhance cell spreading on titanium implants so as to enhance osteointegration (Canullo et al., 2016) and re-osteointegration (Duske et al., 2012).

One viable option to promote the osteoconductivity of bone substitutes and metallic implants is to apply bioactive peptides (Lee et al., 2008; Pountos et al., 2016). An interesting candidate peptide in this respect is histatin-1 (Hst1), a member of a large histidine-rich peptide family that is present in human saliva. Various Hsts have been implicated in numerous protective functions in the mouth, such as detoxification (Yan and Bennick, 1995), antimicrobial activity (Xu et al., 1991; Kavanagh and Dowd, 2004) and inhibition of enamel demineralization (Yin et al., 2003). In our previous studies, we have demonstrated that Hst1 promotes the wound closure of human epithelial cells *in vitro* (Oudhoff et al., 2008, 2009a, b), putatively by the activation of G protein-coupled receptor (GPCR) and extracellular-signal-regulated kinase (ERK), but not p38 MAPK (mitogen-activated protein kinase) signaling pathways (Oudhoff et al., 2008, 2009b). Furthermore, it has been found that Hst1 promotes the adhesion and spreading of epithelial cells onto bio-inert glass, bio-inert substrate (Van Dijk et al., 2015) and on hydroxyapatite and sputtered titanium *in vitro* (Van Dijk et al., 2017a). Meanwhile, recent studies demonstrate that Hst1 also promotes the attachment of osteogenic cells on titanium SLA (sandblasted and acid etched) surfaces (Van Dijk et al., 2017a) as well as their migration (Castro et al., 2019), which suggests a promising application potential of Hst1 for promoting the osteoconductivity of various medical devices. However, the effect of Hst1 on the spreading of osteogenic cells on titanium SLA surfaces remains to be elucidated.

Hitherto, there is no report to systematically investigate the dose-dependent effect of Hst1 on the spreading of osteogenic cells and its potential molecular mechanisms. In the present study, we explored the effects of Hst1 and its truncated variants on the spreading of osteogenic cells, as well as the involvement of cell signaling pathway using specific inhibitors. As model surface it was chosen to use glass cover slips as they are widely adopted to investigate cell behaviors on bio-inert surfaces. Glass coverslips are also transparent and can thus be used to observe both live and fixed cells using light or fluorescent microscopy (Islam et al., 2016; Van Dijk et al., 2017a; Che et al., 2018). In addition, we also investigated the effect of Hst1 on cell spreading on titanium SLA surface — a most commonly used surface for dental implants.

## Materials and Methods

### Cell Culture

Osteogenic cells [MC3T3-E1 mouse pre-osteoblast cell line, subclone 4, CRL-2593, American Type Culture Collection (ATCC)], was cultured in alpha-Minimum Essential Medium (α-MEM) (Gibco, Thermo Fisher Scientific). All media were supplemented with 10% fetal bovine serum (FBS, Thermo Fisher Scientific), 10 units/mL penicillin and 10 μg/mL streptomycin (Invitrogen, Thermo Fisher Scientific). Cells were cultured at 37°C in a moist atmosphere at 5% CO_2_ and routinely tested for the presence of mycoplasm. In all experiments, cells from exponentially growing cultures were used.

### Solid-Phase Peptide Synthesis

All peptides (Table 1) were manufactured by solid-phase peptide synthesis using 9-fluorenylmethoxycarbonyl (Fmoc)-chemistry as described previously (Bolscher et al., 2011; Van Dijk et al., 2015). The peptides were purified by High-Performance Liquid Chromatography (RF-HPLC, Dionex Ultimate 3000, Thermo Scientific, Breda, Netherlands) to a purity of at least 95%. The authenticity was confirmed by mass spectrometry with a Microflex LRF MALDI-TOF (Bruker Daltonik GmbH, Bremen, Germany) as previously described (Bolscher et al., 2011; Van Dijk et al., 2015). During synthesis, part of Hst1 was labeled with the fluorescent dye ATTO-647N (ATTO-TEC GmbH, Siegen, Germany). An equimolar amount of the dye was coupled to the ε-amino group of the side chain of lysine residue number 17 (lys17, K of Hst1 after removal of the specific protective (ivDde)-OH group by hydrazine (2% hydrazine hydrate).

**TABLE 1:** Amino acid sequences of Hst1, Scrambled Hst1 (Scr-Hst1), and Hst1 truncated variants.

Name	Amino acid sequence
Control	No peptide
Scr-Hst1	SDHSRHEEFKPRFHYHGGDYYRGRSKNFYHLEYKDHNH
Hst1 (1–38)	DSHEKRHHGYRRKFHEK HHSHREFPFYGDYGSNYLYDN
Hst1 (12–38)	………………………RKFHEKHHSHREFPFYGDYGSNYLYDN
Hst1 (16–34)	………………………………EKHHSHREFPFYGDYGSNY………
Hst1 (18–34)	………………………………….HHSHREFPFYGDYGSNY………
Hst1 (20–34)	………………………………………SHREFPFYGDYGSNY………
Hst1 (20–32)	………………………………………SHREFPFYGDYGS…………..
Hst1 (20–30)	………………………………………SHREFPFYGDY……………….

*Double underlined K in Hst1 is labeled with a fluorescent molecule ATTO647.*

### Cell Spreading Assay

In this experiment, four groups of three wells each were set up each time in 12-well cell culture plates. Each experiment was repeated at least two times for statistical analysis, so *N* = 6 wells per group. Cells were serum deprived for 24 h, detached using 0.05% trypsin (Gibco), and suspended in culture medium containing 2% FBS to inactivate the trypsin and centrifuged at 200 g for 5 min at room temperature. Next, cells were re-suspended in their prescribed medium without serum and counted using a hemocytometer. Cells were seeded on glass coverslips (diameter, 12 mm, No. 1, VWR, Amsterdam, Netherlands) in 12-wells suspension cell culture plates (Greiner Bio-One, Alphen aan de Rijn, Netherlands) at a density of 6 × 10^4^ cells/well, treated with 0–20 μM of the Hst1 or 10 μM truncated Hst1 or scrambled Hst1. Cells were imaged every 20 min during a 3 h period using an EVOS-FL microscope (Thermo Fisher Scientific) equipped with a LPlanFL PH2 20x using the phase contrast setting or the Cy5 light cube (628/40 and 692/40 nm, excitation and emission filters, respectively). For each well, 6 images were taken randomly with about 90–120 cells per image. Thereafter, we randomly selected 3 images for further analysis by measuring the surface area of cells’ filopodia and lamellipodia (denoted in Supplementary Figure 1 with the red circle) using a point-counting method (Gundersen and Jensen, 1987; Supplementary Figure 1). The surface area of cell was considered to quantitatively represent cell spreading.

### Uptake and Localization of Hst1 in Osteogenic Cells

To analyze the uptake and localization of fluorescently labeled Hst1 (F-Hst1) by osteogenic cells, cells from a semi-confluent cell culture were transferred into a 48-wells plate at a density of about 3.5 × 10^4^ cells/well and cultivated at 37°C for at least 24 h. Subsequently, the cells were washed once with DPBS (Dulbecco’s PBS, Gibco) after which serum-free medium was added. Two micrometer F-Hst1 was added and after a 1 h period, the cells were washed four times with DPBS containing 0.9 mM Ca^2+^ and 0.5 mM Mg^2+^. As a negative control, incubations without F-Hst1 were included. The cells were studied by the EVOS-FL microscope with a 20x objective with a phase contrast setting and a “Cy-5 light cube” with a 628/40 excitation filter and a 692/40 nm emission filter. Digital photographs were recorded by a computer integrated in the microscope.

To more precisely observe the uptake and localization of F-Hst1, the cells were further studied using a LEICA TCS SP8 confocal laser scanning microscopy (CLSM) system as previously described (Ma et al., 2020). Before incubation with F-Hst1, cell nuclei and membrane were stained with NucBlue^TM^ live cell stain (Life Technologies, Grand Island, NY) and PKH67GL (Sigma–Aldrich, MO, United States) respectively, following manufacturer’s instructions.

### Signaling Pathways for the Effects of Hst1

In our previous studies, we have demonstrated that Hst1 promotes the wound closure of human epithelial cells *in vitro* (Oudhoff et al., 2008, 2009a, b), putatively by the activation of GPCR and ERK1/2, but not p38 MAPK signaling pathways (Oudhoff et al., 2008, 2009b). To investigate the role of potential signaling pathways in the effects of Hst1 on the spreading of osteogenic cells, we applied the following signaling pathway-specific inhibitors of ERK1/2 (U0126, 10 μM; LC Laboratories, Woburn, MA, United States), GPCR (pertussis toxin (PTx), 200 ng/mL; LC Laboratories, Woburn, MA, United States), and p38 MAPK (SB203580, 10 μM; LC Laboratories, Woburn, MA, United States) using the cell spreading model on bio-inert glass.

### Cell Spreading on Titanium SLA Surface

Commercially titanium (purity > 99.6%, 125 μm in thickness, ADVENT, United Kingdom) foil was cut into round discs (5 mm in diameter). To make SLA surface, the titanium discs were first sandblasted with large corundum grits. Thereafter, the discs were cleaned ultrasonically in alcohol and dH_2_O for 15 min respectively, and then acid etched with a mixture of 37% HCL 25 ml, 98% H_2_SO_4_ 25 mL and 50 mL dH_2_O solution at 60°C for 22 min in a stirring condition (100 rpm). Finally, the discs were cleaned ultrasonically in dH_2_O for 15 min three times and dried overnight in oven. These discs were thereafter autoclaved for sterilization and kept in sterile condition. Cell spreading on titanium SLA surface was performed as described in the section of cell spreading assay. 1.5 h after seeding, cells on titanium discs were fixed, dehydrated and stained with DAPI and FITC-Phalloidin to stain nuclei and cell skeleton respectively. Fluorescent micrographs were randomly taken using a fluorescent microscope (Olympus BX61) with Excitation/Emission wavelengths (nm) of 358/461 and of 496/516, respectively. On the micrographs, spreading surface of each cell was estimated using the abovementioned point-counting method. More than 30 cells per group were calculated.

### Statistical Analysis

**FIGURE 1:**
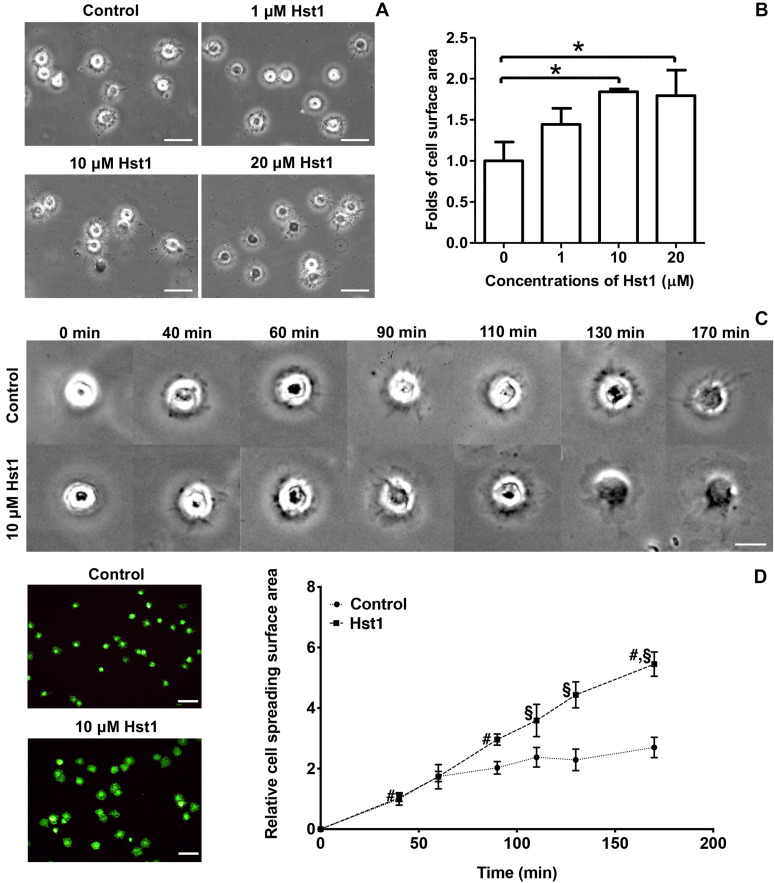
Hst1 promoted the spreading of osteogenic cells on glass surfaces. **(A)** Light micrographs depicting the spreading of osteogenic cells in the absence or presence 1, 10, and 20 μM Hst1. Bar = 50 μm. **(B)** Folds of cell spreading surface area in the absence or presence or of 1, 10, and 20 μM Hst1. Data are shown as mean ± SD (*n* = 6). *p < 0.05 **(C)** Light micrographs of the spreading of osteogenic cells in the absence or presence of 10 μM Hst1 on different time points. **(D)** Fluorescent micrographs depicting the spreading of osteogenic cells (stained with FITC-Phalloidin) in the presence or absence of 10 μM Hst1 on bio-inert glass surface. Time-dependent cell spreading surface area (expressed in folds with the value of the control group at first time point as 1) in the presence or absence of 10 μM Hst1. Data were shown as mean ± SD (*n* = 6). ^§^ *p* < 0.05 indicating a significant difference compared with the values in the control group at the same time point; ^#^*p* < 0.05 indicating a significant difference compared with the value in the same treatment group at the earlier time point. Bar = 50 μm.

All experiments were carried out at least three times and in quadruple. Data were plotted using Graphpad Prism (Graphpad Software version 6.0, La Jolla, CA, United States), analyzed using Student’s *t*-test and one- way ANOVA with Bonferroni’s *post hoc* test for multiple comparisons. For the data from different groups at different time points in Figure 1D, we used two-way ANOVA to analyze the data with Bonferroni’s *post hoc* test for multiple comparisons. Results were reported as mean ± SD. A value of *P* < 0.05 was considered as statistical significance. ^∗^*p* < 0.05; ^∗∗^*p* < 0.01; ^∗∗∗^*p* < 0.001.

## Results

### Hst1 Promoted Spreading of Osteogenic Cells on Bio-Inert Glass

We first evaluated the dose-dependent effect of Hst1 on cell spreading. The presence of Hst1 promoted the spreading of osteogenic cells with larger lamellipodia and more filopodia in comparison with the control (no Hst1) (Figure 1A). Compared to the control, treatment with Hst1 at concentrations of 10 and 20 μM resulted in significantly larger spreading surface area (Figure 1B) 170 min post incubation. Thereafter, we assessed the dose-dependent effects of Hst1 on cell spreading. Cell treated with Hst1 for > 90 min, showed significantly higher spreading than the control (untreated cells) (Figure 1C). The spreading areas in Hst1 group were 1.46, 1.51, 1.93, and 2.02 times those in control group at 90, 110, 130, and 170 min, respectively (Figure 1D).

### Truncated Hst1 Variants Did Not Improve the Spreading of Osteogenic Cells on Bio-Inert Glass

**FIGURE 2:**
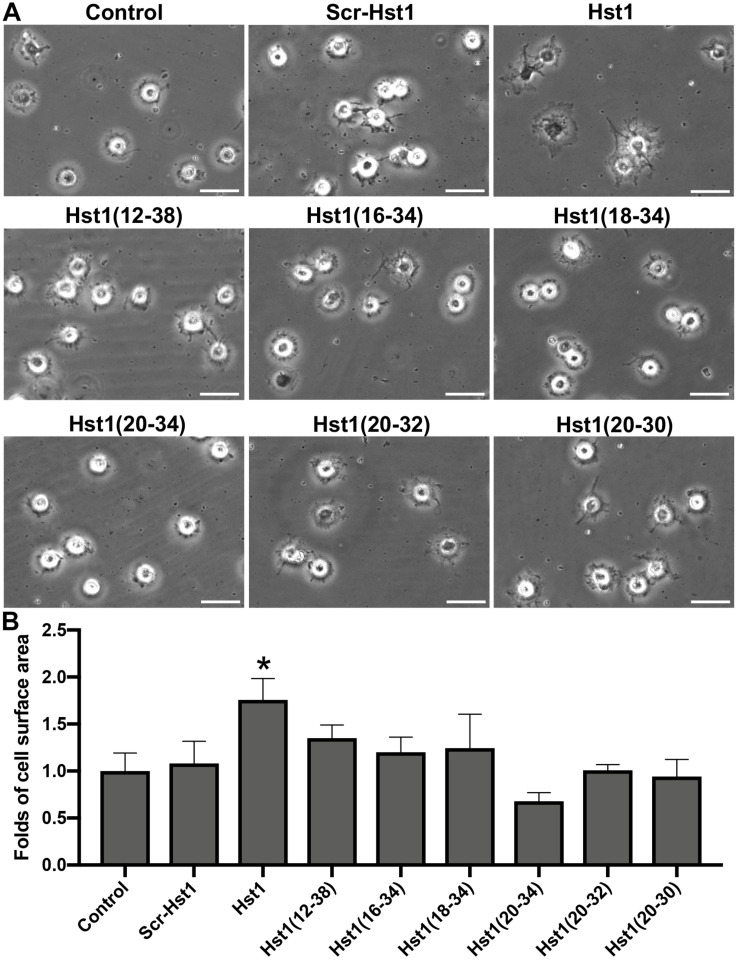
The effect of truncated Hst1 variants on the spreading of osteogenic cells. **(A)** Light micrographs depicting the spreading of osteogenic cells in the presence or absence of scrambled Hst1 and 6 truncated variants of Hst1 on glass surfaces. Bar = 50 μm. **(B)** Folds of cell spreading surface area in the presence or absence of scrambled Hst1 and 6 truncated variants of Hst1 on glass. Data are shown as mean ± SD (*n* = 6). *p < 0.05.

Scrambled Hst1 did not promote spreading of osteogenic cells compared to the control. In order to determine the minimal domain of Hst1 required for cell spreading on bio-inert glass, we tested 6 truncated variants of Hst1. Since the *in vitro* wound-healing properties of Hst1 (amino acids 12–38) appeared comparable to those of the parent peptide Hst1 (Oudhoff et al., 2008), this peptide was used as a starting point for mapping the minimal active domain. To map the minimal active domain responsible for the cell spreading, a number of truncated variants of Hst1 were synthesized (encompassing amino acids 12–38, 16–34, 18–34, 20–34, 20–32, and 20–30) as previously reported (Oudhoff et al., 2009a). The relative spreading area of osteogenic cells under the stimulation of Hst1 (1–38), Hst1 (12–38), Hst1 (16–34), Hst1 (18–34), Hst1 (20–34), Hst1 (20–32), and Hst1 (20–30) were 1.76 ± 0.23, 1.35 ± 0.14, 1.20 ± 0.16, 1.24 ± 0.36, 0.68 ± 0.09, 1.01 ± 0.06, and 0.94 ± 0.18, respectively (Figure 2). However, except for Hst1 (1–38), none of these variants, significantly promoted the spreading of osteogenic cells on glass surface in comparison with the control (1.00 ± 0.19) (Figure 2).

### Uptake and Localization of Hst1 by Osteogenic Cells

**FIGURE 3:**
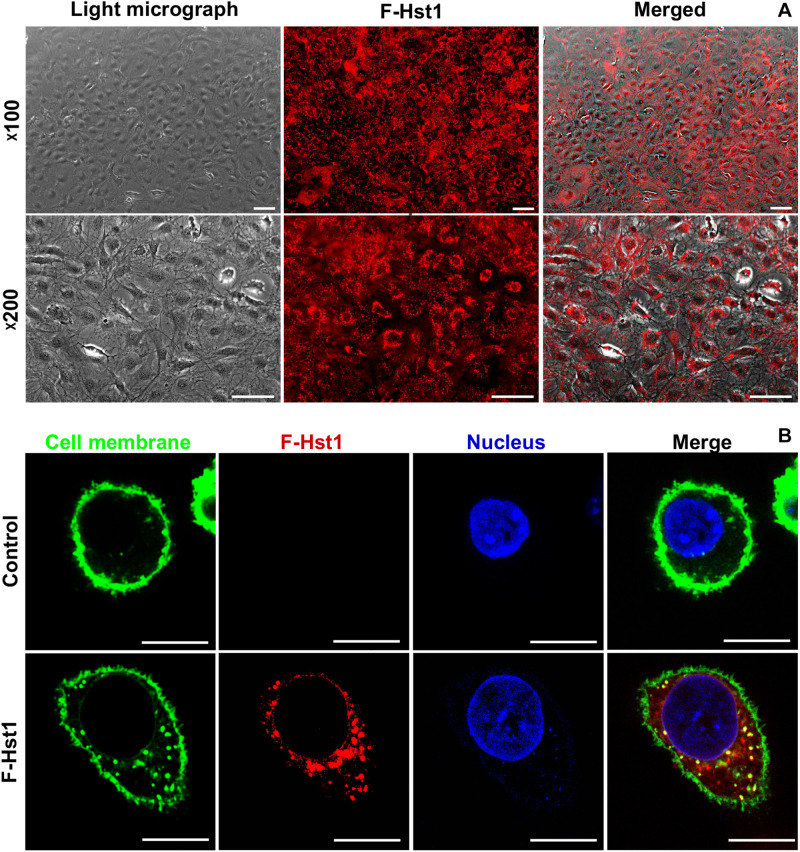
The uptake and distribution of F-Hst1 in osteogenic cells. **(A)** Light (left column) and fluorescent (middle column) micrographs depicting the uptake of fluorescently labeled Hst1 (F-Hst1) (middle column) by osteogenic cells (left column) in lower (upper row) and higher (lower row) magnification. Bar = 100 μm. **(B)** Cellular uptake of the fluorescence (ATTO-647N)-labeled Hst1 (F-Hst1). CLSM images of F-Hst1 variants (in red) were taken up by osteogenic cells; cell membrane are stained in green; nuclei are stained in blue; Bar = 20 μm.

In order to study whether Hst1 is also taken up by osteogenic cells, MC3T3-E1 cells were incubated with F-Hst1 and microscopically analyzed. After a 1 h incubation, an intracellular bright, granular labeling pattern was observed in the vicinity of nuclei (Figure 3A). CLSM micrographs showed that F-Hst1 (red) distributed between cell membrane (green) and nucleus (blue), while in the control group (no Hst1) red fluorescence was detected in intracellular space (Figure 3B).

### Effects of Inhibitors of ERK1/2, P38 and GPCR on Hst1-Induced Cell Spreading

**FIGURE 4:**
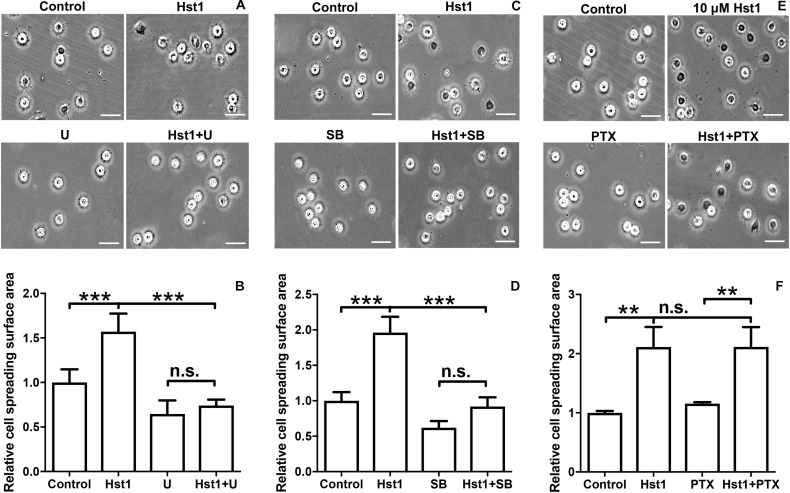
The effects of signaling pathway inhibitors on Hst1-induced spreading of osteogenic cells. Light micrographs depicting the spreading of osteogenic cells that were treated with **(A)** extracellular-signal-regulated kinase (ERK) signaling (10 μM U0126), **(C)**: p38 signaling (10 μM SB203580) and **(E)** G protein-coupled receptor (GPCR) (200 ng/mL Pertussis toxin, PTx). Bar = 50 μm. Relative spreading surface area of osteogenic cells that were treated with Hst1 with or without the pretreatment with the inhibitors of **(B)** extracellular-signal-regulated kinase (ERK) signaling (10 μM U0126), **(D)** p38 signaling (10 μM SB203580) and **(F)** G protein-coupled receptor (GPCR) (200 ng/mL Pertussis toxin, PTx). Data are shown as mean ± SD (*n* = 6). **p < 0.01, ***p < 0.001.

To reveal the potential involvement of signaling pathways in Hst1-induced spreading of osteogenic cells, we analyzed the effects of the ERK1/2 inhibitor (U0126), p38 MAPK inhibitor (SB203580) and PTx (an inhibitor of GPCR) on the Hst1-induced cell spreading. U0126 (10 μM) or SB203580 (10 μM) completely abolished the stimulating effects of Hst1 on the spreading of osteogenic cells (Figures 4A–D). In contrast, PTx did not show any significant effect (Figures 4E,F). The inhibitors had no effect on the basal cell spreading in the absence of Hst1.

### Hst1 Enhanced the Osteogenic Cell Spreading on Titanium SLA Surface

**FIGURE 5:**
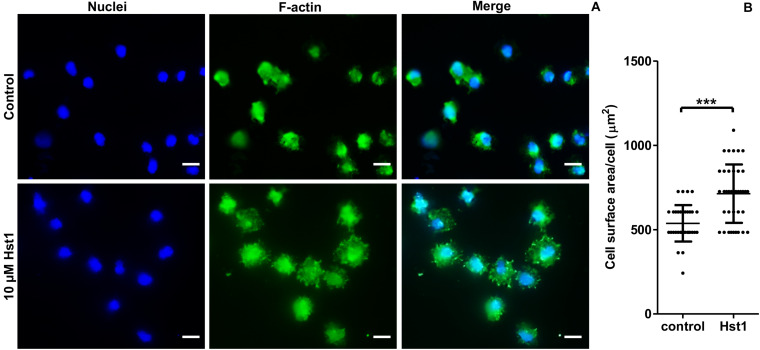
Hst1 promoted the spreading of osteogenic cells on titanium SLA surfaces. Fluorescent micrographs **(A)** depicting the spreading of osteogenic cells that were stained by Dapi (for nuclei) (left column) and by FITC-Phalloidin (for F-actin) (middle column) Bar = 20 μm. **(B)** Graph depicting the quantitative analysis of the spreading of osteogenic cells. Data are shown as mean ± SD (*n* = 32). ***p < 0.001.

In order to assess the promoting effect of Hst1 on osteogenic cells on titanium plates, MC3T3-E1 cells were seeded on the titanium plates with SLA surfaces in the absence or presence of 10 μM Hst1. To enable quantification of the cell-surface area by fluorescence microscopy, cellular actin was fluorescently labeled using FITC-Phalloidin (Figure 5A). Quantitative analysis indicated that the surface area per cell in the group of 10 μM Hst1 (713.26 ± 172.94 μm^2^) was significantly higher than that without Hst1 (537.38 ± 108.19 μm^2^) (Figure 5B).

## Discussion

The adhesion, spreading and migration of osteogenic cells on medical devices are indispensable for effective bone formation of LVBD and osteointegration of implants (Deligianni, 2014; Zhang et al., 2015). In this study, we demonstrated that Hst1 significantly promoted the spreading of osteogenic cells on bio-inert glass in dose- and time-dependent assays. It appeared that such effect of Hst1 was associated with ERK1/2 and p38 signaling as their specific inhibitors counteracted the Hst1 stimulation. We also showed that Hst1 significantly promoted the spreading of osteogenic cells on SLA titanium surface. Therefore, Hst1 bears an application potential to promote osteoconductivity, so as to enhance bone healing and osteointegration.

It is well established that cell-substrate contact area can determine cell activities, such as proliferation, quiescence or apoptosis (McGrath, 2007). When plated on glass, the cells go through three major phases from the unsuspended state to the flat and polarized state: (1) initial attachment; (2) a rapid increase in cell spreading area through depletion of membrane reservoirs; and (3) a slower spreading phase that includes periodic protrusion/retraction of the cell edge and an increase in membrane area (Dubin-Thaler et al., 2004; Giannone et al., 2004). The advantage of using the model system of cell spreading from the unattached state is that it is highly reproducible and the stages that the cell undergoes can be characterized and quantified in a time-dependent manner (Wolfenson et al., 2014). There exist many experimental methods to quantitatively evaluate cell spreading, such as the determination of relative cell spreading and calculation of the so called cell index (Van Dijk et al., 2015, 2017a). The later parameter qualifies the impedance of cells that proportionally correlate to, but not directly show, cell spreading extent. In contrast, in the current study, we adopted a point-counting method (Cruz-Orive and Weibel, 1990) to measure the surface area of spreading cells, which could directly reflect the newly formed cell-substrate contact area.

Hst1 has already been shown to be efficacious in promoting the adhesion, spreading and migration of various epithelial cells from skin (Oudhoff et al., 2009a), mucosal (Oudhoff et al., 2008, 2009b), gingival (Van Dijk et al., 2015, 2017a) and corneal origin (Shah et al., 2017). Hst1 bears a very broad pharmaceutical dosage. Hst1 is associated with no cytotoxicity and significantly enhanced metabolic activity of human corneal epithelial cells within 200 μM (Shah et al., 2017). In our recent study, we also show that 10 μM Hst1 significantly promotes metabolic activity of human oral mucosal epithelial cells, skin keratinocytes and gingival fibroblasts (Ma et al., 2020). These findings indicate the good biocompatibility of Hst1. It remains unclear how Hst1 influences cellular metabolic activity. Our recent study shows that Hst1 can be quickly internalized and accumulate in cytoplasm (Ma et al., 2020). In the present study, we adopted CLSM and, for the first time, clearly showed the uptake of Hst1 by pre-osteoblasts. After being taken up, Hst1 is quickly targeted to mitochondria and endoplasmic reticulum (ER) (Ma et al., 2020), which suggests a potential role of mitochondria-ER contact in the modulating effects of Hst1 on cellular metabolic activity. However, hitherto, the intracellular signaling pathways and genes activated by Hst1 remains to be elucidated. Further studies should be performed to clarify these issues.

Consistent with these reports, it was observed that 10 μM of Hst1 optimally promoted the spreading of osteogenic cells *in vitro* 60 min post incubation. The time-dependent course of Hst1’s effect was consistent with the previous findings on epithelial cells that the surface of fibroblasts increased remarkably after 60 min treated with Hst1 when compared to the control (Van Dijk et al., 2015, 2017a). Further publications also reported that Hst1 could promote endothelial cell adhesion, migration, and angiogenesis (Torres et al., 2017; Van Dijk et al., 2017b). Hst1 was also demonstrated to promote the attachment of osteogenic cells on the both sputtered smooth titanium surface and SLA surfaces (Van Dijk et al., 2017a). In the current study, we showed that Hst1 could also promote the spreading of osteogenic cells on both bio-inert glass surfaces as well as on titanium SLA surfaces. These findings suggested that Hst1 could improve cell-biomaterial interaction so as to promote bone healing and implant osteointegration. Furthermore, Hst1 is also shown to counteract the cytotoxic and anti-migratory effects of zoledronic acid on MC3T3-E1 osteogenic cells and endothelial cells (Castro et al., 2019). Zoledronic acid, the most frequent agent associated with bisphosphonate-related osteonecrosis of the jaw, has been reported as cytotoxic for bone and vascular cells (Aghaloo et al., 2015). It has been shown that Hst1 can protect cells from different adverse conditions by decreasing cell apoptosis (Aghaloo et al., 2015; Huang et al., 2018). These findings suggested that Hst1 has a great application potential in various bone diseases.

Titanium is widely used as implant material in case of e.g., cervical prosthetics, joint prosthetics, mini-plates, mini-screws, and dental implants (Giannasi et al., 2018). Continuous efforts have been made to enhance cell spreading on titanium implants with an aim to facilitate osteointegration (Canullo et al., 2016) and re-osteointegration (Duske et al., 2012). For this purpose, various peptides have also been attempted. For example, P15, a synthetic 15 amino acid peptide mimicking the cell-binding domain within the alpha-1 chain of human collagen, can significantly promote cell attachment, spreading and osteogenic gene expression (Liu et al., 2012). Peptide derived from another cell matrix laminin also promoted cell attachment of human osteoblast-like cells (Min et al., 2013). To exert such functions, most of these peptides still needs to be pre-attached onto the surface of titanium (Liu et al., 2012; Min et al., 2013). Different from the application pattern of these peptides, we showed that Hst1 could promote the attachment of osteogenic cells without the needs of pre-attachment to titanium surfaces. In this study, we investigated the effect of Hst1 on osteogenic cell spreading on titanium SLA surface, the most commonly used surface for dental implants. Consistent with the previous conclusion (Van Dijk et al., 2017a), we showed that Hst1 significantly enhanced the spreading of osteogenic cells on titanium SLA surfaces when compared to the control group. In our recent study, we showed that Hst1 could be quickly taken up and distribute in the vicinity of surrounding nuclei within epithelial cells and fibroblasts, which suggested an important role of subcellular targets (mitochondria and endoplasmic reticulum) in Hst1’s functions (Ma et al., 2020). In this study, our data obtained using fluorescent microscopy showed that F-Hst1 was taken up by osteogenic cells and distributed intracellularly. CLSM results corroborated that Hst1 was distributed in the vicinity of the nucleus. These findings suggested that Hst1 did not act as an intermediate (coating) between substrate and cell, but rather induces a cellular response. With these properties, Hst1 can be used as convenient stimulatory molecule to promote osteointegration of titanium implants.

Hitherto, it is still unclarified by which exact molecular mechanism Hst1 promotes cell spreading. Our previous studies suggest that the promoting effect of Hst1 on the migration of epithelial cells is mediated by the GPCRs-ERK1/2 pathway, but not by the p38 pathway (Oudhoff et al., 2009a). Our recent study shows that co-administration of Hst1 with all-trans retinoic acid additively stimulates the spreading and osteogenicity of preosteoblasts on bio-inert glass surfaces *in vitro*, which can be abolished by specific inhibitors of retinoic acid receptors α (but not β or γ) (Sun W. et al., 2020). However, the signaling pathways accounting for the Hst1’s effect on the pre-osteoblasts’ adhesion remains to be clarified. In this study, we found that U0126, an inhibitor of ERK1/2 pathway, significantly inhibited the effect of Hst1. However, also the p38 inhibitor SB203580 abolished the effect of Hst1, suggesting that in the present system, both ERK1/2 and p38 pathways wound to be involved in mediating the effect of Hst1. This is incongruent with a previous study in which it was found that inhibition of ERK1/2, but not of p38, abrogated the stimulatory effects of Hst1 (Oudhoff et al., 2009a). Furthermore, in that study, it is found that Hst1 (20–32) (SHREFPFYGDYGS) is the minimal domain of Hst1 that promotes the migration of epithelial cells (Oudhoff et al., 2009a). Such a study is highly important to identify a minimal functional domain. Furthermore, a much shorter peptide would be very meaningful for its pharmaceutical application due to a significantly lower production cost. In the present study, we, for the first time, showed the effects of truncated Hst1 variants on the spreading of pre-osteoblasts. Inconsistent with the previous finding, our present study showed that none of truncated Hst1 variants had any effect on osteogenic cell spreading. Further studies are needed to clarify these apparent discrepancies, which may be caused by the use of different cell types (epithelial vs. osteogenic cells) from different species (human vs. mouse) in these studies. Although our data suggest the roles of ERK and p38 signaling in the promoting effects of Hst1 on cell adhesion, it remains unclear how these two signaling pathways interact with the cell skeleton-modulating signaling cascades, such as phosphatidylinositol 3 kinases, ERK1/2, 5’-AMP-activated protein kinase, members of the Rho GTPase family (RhoA, Rac, Cdc42), Rho guanine nucleotide exchange factors, Rho GTPase activating proteins, and Rho guanine nucleotide dissociation inhibitors (Moujaber and Stochaj, 2020). Further studies should be performed to clarify how Hst1 interacts with these signaling pathways.

On the other hand, ERK1/2 and p38 are proved to be involved in osteogenic differentiation (Guo and Wu, 2012). Under the stimulation of bone morphogenetic protein 2 (BMP2), the inhibition of p38 by SB203580 was associated with a significantly decreased alkaline phosphatase activity (an early osteogenic differentiation marker) and osteocalcin (OCN, a late osteogenic differentiation marker) in MC3T3-E1 pre-osteoblasts (Guicheux et al., 2003), while ERK signaling was barely activated, which indicates the importance of p38 but not ERK signaling in BMP2-induced osteogenic differentiation. On the other hand, icaritin, a hydrolytic product of icariin from the genus Epimedium, can significantly upregulate ERK and p38 signaling in its-induced osteogenic differentiation in MC3T3-E1 pre-osteoblasts. Furthermore, p38 antagonist SB203580 and ERK1/2 antagonist PD98059 markedly inhibit the icaritin-induced the mRNA expression of ALP, COL1 (encoding collagen type I), OCN and OPN (encoding osteopontin) (Wu et al., 2017). These results suggest that different ligands may stimulate different patterns of signaling. Since ERK and p38 seems to be involved in Hst1’s effect, question may be raised whether Hst1 can promote *in vitro* osteogenic differentiation and *in vivo* osteogenesis, thereby contributing to osteoinductivity—another important property to heal LVBD and facilitate osteointegration. Hitherto, this issue is rarely reported. In our recent study, we show that Hst1 alone does not promote ALP activity (Sun W. et al., 2020). Additionally, our ongoing study shows that Hst1 does not further enhance BMP2-induced *in vitro* extracellular mineralization of MC3T3-E1 (data not shown). Therefore, although ERK and p38 signaling are involved in Hst1’s effects on cell adhesion and spreading, they don’t directly lead to osteogenic differentiation at least in MC3T3-E1 pre-osteoblasts. All these data suggest that Hst1 does not enhance osteogenic differentiation, thus bearing no osteoinductivity. On the other hand, in our recent *in vivo* study, Hst1 significantly promotes angiogenesis and osteogenesis under the stimulation of BMP2 in an ectopic bone induction model (Sun P. et al., 2020). Such an effect of Hst1 on osteogenesis may be attributed to the stimulating effect of Hst1 on angiogenesis (Torres et al., 2017) that favors bone regeneration (Grosso et al., 2017). However, caution should be always taken to completely exclude the direct stimulating effects of Hst1 on osteogenic differentiation because only a mouse partially osteogenically-committed cell line has been examined. Human primary mesenchymal stem cells, such as bone marrow stem cells, should be used in future study to further investigate the effects of Hst1 on osteogenic differentiation.

## Data Availability Statement

All datasets presented in this study are included in the article/Supplementary Material.

## Author Contributions

GW, HL, and EV: conceptualization. WS, GW, and DM: investigation. GW and JB: resources. WS and GW: formal analysis. KN and FB: data curation. WS, FB, and GW: writing—original draft preparation. PS, WS, DM, FB, JB, KN, HL, GW, and EV: editing. WS, GW, PS, HL, and EV: funding acquisition. All authors contributed to the article and approved the submitted version.

## Conflict of Interest

The authors declare that the research was conducted in the absence of any commercial or financial relationships that could be construed as a potential conflict of interest.
