# Supplementary material for: Human Salivary Histatin-1 Promotes Osteogenic Cell Spreading on Both Bio-Inert Substrates and Titanium SLA Surfaces
Source: Front Bioeng Biotechnol. 2020 Oct 23;8:584410. doi: 10.3389/fbioe.2020.584410 (PMC7649783; doi:10.3389/fbioe.2020.584410)

Supplementary Material

# Supplementary Figures 1

Graph depicting a point-counting method to measure the surface area of cell spreading. The grid was randomly put on the light micrographs of cells during spreading for the point-counting method. The filopodia and lamellipodia (red arrow) was included for calculating the cell spreading area with the exclusion of the relatively constant peri-nuclear area (within red dot circle). Bar = 50 μm.


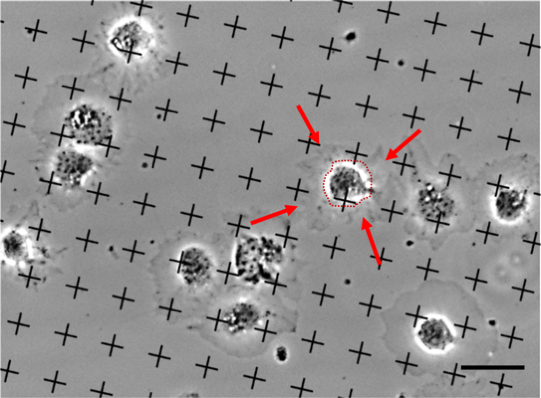

Supplement: Supplementary file 1 [file Data_Sheet_1.docx]
